# Erythropoietin Receptor Antagonist Suppressed Ectopic Hemoglobin Synthesis in Xenografts of HeLa Cells to Promote Their Destruction

**DOI:** 10.1371/journal.pone.0122458

**Published:** 2015-04-15

**Authors:** Yoshiko Yasuda, Mitsugu Fujita, Eiji Koike, Koshiro Obata, Mitsuru Shiota, Yasushi Kotani, Terunaga Musha, Sachiyo Tsuji-Kawahara, Takao Satou, Seiji Masuda, Junko Okano, Harufumi Yamasaki, Katsumi Okumoto, Tadao Uesugi, Shinichi Nakao, Hiroshi Hoshiai, Masaki Mandai

**Affiliations:** 1 Departments of Obstetrics and Gynecology, Kinki University Faculty of Medicine, Osakasayama, Osaka, Japan; 2 Departments of Microbiology, Kinki University Faculty of Medicine, Osakasayama, Osaka, Japan; 3 Department of Obstetrics and Gynecology, Nara Hospital Kinki University Faculty of Medicine, Ikoma, Nara, Japan; 4 Department of Gynecological Oncology, Kawasaki Medical University, Kurashiki, Okayama, Japan; 5 Department of Gynecology, Medicalcourt Hachinohe West Hospital, Hachinohe, Aomori, Japan; 6 Departments of Immunology, Kinki University Faculty of Medicine, Osakasayama, Osaka, Japan; 7 Departments of Pathology, Kinki University Faculty of Medicine, Osakasayama, Osaka, Japan; 8 Laboratory of Molecular Biology of Bioresponse, Graduate School of Biostudies, Kyoto University, Kyoto, 606–8502, Japan; 9 Division of Anatomy and Cell Biology, Shiga University of Medical Science, Otsu, Shiga, Japan; 10 Lifescience Institute, Kinki University Faculty of Medicine, Osakasayama, Osaka, Japan; 11 Departments of Anesthesiology, Kinki University Faculty of Medicine, Osakasayama, Osaka, Japan

## Abstract

The aim of this study is to explore a cause-oriented therapy for patients with uterine cervical cancer that expresses erythropoietin (Epo) and its receptor (EpoR). Epo, by binding to EpoR, stimulates the proliferation and differentiation of erythroid progenitor cells into hemoglobin-containing red blood cells. In this study, we report that the HeLa cells in the xenografts expressed ε, γ, and α globins as well as myoglobin (Mb) to produce tetrameric α2ε2 and α2γ2 and monomeric Mb, most of which were significantly suppressed with an EpoR antagonist EMP9. Western blotting revealed that the EMP9 treatment inhibited the AKT-pAKT, MAPKs-pMAPKs, and STAT5-pSTAT5 signaling pathways. Moreover, the treatment induced apoptosis and suppression of the growth and inhibited the survival through disruption of the harmonized hemoprotein syntheses in the tumor cells concomitant with destruction of vascular nets in the xenografts. Furthermore, macrophages and natural killer (NK) cells with intense HIF-1α expression recruited significantly more in the degenerating foci of the xenografts. These findings were associated with the enhanced expressions of nNOS in the tumor cells and iNOS in macrophages and NK cells in the tumor sites. The treated tumor cells exhibited a substantial number of perforations on the cell surface, which indicates that the tumors were damaged by both the nNOS-induced nitric oxide (NO) production in the tumor cells as well as the iNOS-induced NO production in the innate immune cells. Taken together, these data suggest that HeLa cells constitutively acquire ε, γ and Mb synthetic capacity for their survival. Therefore, EMP9 treatment might be a cause-oriented and effective therapy for patients with squamous cell carcinoma of the uterine cervix.

## Introduction

Erythropoietin (Epo) is a hypoxia-inducible cytokine that regulates erythropoiesis. Epo binds to its receptor (EpoR) on erythroid progenitors to support their survival and stimulate their proliferation and differentiation into hemoglobin (Hb)-containing erythrocytes [1]. Globin synthesis is initiated in the colony forming unit of erythroids (CFU-E) [2]. Hb contains 4 subunit proteins that consists of two globin peptide chains: adult Hb (HbA) α_2_β_2_, embryonic Hb (HbE) α_2_ε_2_, and fetal Hb (HbF) α_2_γ_2_ [3]. Each peptide chain carries a heme prosthetic group bound non-covalently. In addition to physiological erythropoiesis, ectopic Hb synthesis (ε and β) occurs temporarily in the early mouse embryo proper with the surrounding decidual cells at the developmental stage prior to feeding vessel establishment [4]. In the human decidua, ε, γ, β and α as well as cytoglobin and myoglobin (Mb) are expressed prior to the establishment of the feto-placental circulation [5]. In these sites, Epo co-regulates the expression of a globin and heme-synthesizing enzyme, non-erythroid δ-aminolevurinate synthase (ALAS-N) [4, 5], through phosphatidylinositol-3-kinase / protein kinase B (PI3K/AKT) pathway [5]. These ectopic hemoproteins are expressed temporarily and rigidly controlled by the oxygen demands *in situ*.

In addition to erythropoiesis, Epo play pivotal roles in other biological events such as tumor promotion, angiogenesis, and innate immune responses. From the point on that we first reported that normal and malignant female reproductive organs express Epo and EpoR mRNA [6, 7], there has been accumulating evidence that the Epo-EpoR pathway is substantially involved in cancer progression [8–10]. The Epo-EpoR pathway is also involved in angiogenesis due to the expression of EpoR on vascular endothelial cells [11, 12]. Particularly in cervical, uterine, and ovarian cancers, deprivation of Epo signaling destructs the angiogenesis in the tumor microenvironment [6, 13]. In addition, Epo mimetic peptide 9 (EMP9) has been shown to destruct not only tumor cells (melanoma and stomach choriocarcinoma) but also relevant angiogenesis *in vivo* [14]. Here, the EMP9 is one of the 25 derivatives of the synthetic peptide EMP1, which binds to human EpoR to support the proliferation of Epo-responsive cells. In contrast, EMP9 has been shown not to activate the human EpoR-associated downstream events [15]. Therefore, EMP9 acts as an EpoR antagonist [14]. Regarding the involvement of Epo-EpoR pathway in immunity, it has not been studied in depth except for macrophages and dendritic cells, both of which express EpoR [16, 17]. Nevertheless, detailed mechanisms still remain unclear as to how the Epo-EpoR pathway is involved in tumorigenesis as well as tumor-associated microenvironment such as angiogenesis and immune responses.

Nitric oxide (NO) is a pleiotropic regulator, critical to numerous biological processes, including vasodilatation, neurotransmission and macrophage-mediated immunity [18]. The family of nitric oxide synthases (NOS) comprises neuronal NOS (nNOS), endothelial NOS (eNOS), and inducible NOS (iNOS). Generally, nNOS and eNOS are activated in a Ca^2+^-dependent manner [19]. In parallel, eNOS can be activated through AKT signaling pathway, which leads to the enhanced NO production in a Ca^2+^-independent manner [20, 21]. In contrast, iNOS is transcriptionally regulated by surrounding environment such as cytokines (IFN-γ, IL-1β, TNF-α, etc) and/or oxidative stress including hypoxia [19]. Various studies have shown that all three isoforms can be involved in promoting or inhibiting the etiology of cancer. NOS activity has been detected in tumor cells of various histogenetic origins and has been associated with tumor grade, proliferation rate and expression of important signaling components associated with cancer development such as the oestrogen receptor. High levels of NOS expression (for example, generated by activated macrophages) may be cytostatic or cytotoxic for tumor cells, whereas low level activity can have the opposite effect and promote tumor growth. In particular, uterine cervical cancers are known to express iNOS at high levels [22], which suggests that iNOS might be a useful prognositic marker for this type of cancer [23].

Squamous cell carcinoma (SCC) of the uterine cervix is the second most common gynecological malignancy [24]. It develolps in young women largely due to the persistent infection of human papilloma virus (HPV), and the HPV-derived DNA is discernible in many cervical malignancies. Two oncogenes of HPV type 16 (E6 and E7) are prerequisites for the immortalization and malignant transformation of cervical keratinocytes [25]. In particular, the E6 protein binds to the tumor suppresser protein p53, which induces ubiquitin-mediated degradation of the p53 complex and inactivates tumor suppression [26]. Therapeutic strategies of SCC vary from simple hysterectomy to other specific surgery depending on the stage of the disease and the patient factors [27]. However, metastatic and recurrent of SCC still remains as a severe clinical problem. To these patients, cisplatin-based combination chemotherapy such as cisplatin plus topotecan [28] and cisplatin plus paclitaxel [29] is currently recommended although the outcome is still poor. Therefore, there is a great demand for new cause-oriented therapeutic strategies. During the extensive growth of cervical cancers, a hypoxic condition is known to exist in the cervical lesions [30]. In this regard, Epo, one of hypoxia inducible cytokines, has been reported to play a role in the development of SCC [31]. Indeed, 88.9–92% of SCC biopsy samples showed EpoR expressions [32, 33]. Consistently, the HeLa, SiHa, and C33 cervical cancer cell lines have been reported to express Epo and EpoR [34]. Moreover, HIF-1α protein is expressed under normoxia in HeLa cells, which may contribute to the enhancement of the Epo-EpoR pathway [35]. We have already reported that deprivation of the Epo signal by an anti-Epo antibody, soluble form of EpoR (sEpoR) capable of binding to Epo, or the EpoR antagonist EMP9 results in the destruction of the female malignancies described above through the induction of apoptosis and suppressed growth of tumor cells and the destruction of capillary endothelial cells [6, 13, 14, 36]. Based on these findings, we thought that the HeLa-based system appears to be the most suitable to investigate the significance of the Epo-EpoR pathway in cervical SCC-associated tumor microenvironment particularly in vivo.

In this study, we report that the HeLa cells in the xenografts expressed ε, γ, and α globins as well as Mb to produce tetrameric α_2_ε_2_ and α_2_γ_2_ and monomeric Mb, most of which were significantly suppressed with an Epo antagonist EMP9. Western blotting revealed that the EMP9 treatment inhibited the AKT-pAKT, MAPKs-pMAPKs, and STAT5-pSTAT5 signaling pathways. Moreover, the treatment induced apoptosis of the tumor cells and inhibited angiogenesis in the xenografts. These findings were associated with the enhanced expressions of nNOS in the tumor cells and iNOS in macrophages and NK cells in the tumor sites. The treated tumor cells exhibited a substantial number of perforations on the cell surface, which indicates that the tumors were damaged by both the nNOS-induced NO production in the tumor cells as well as the iNOS-induced NO production in the innate immune cells. Taken together, the present results suggest that HeLa cells constitutively acquire ε, γ and Mb synthetic capacity for their survival. Therefore, EMP9 treatment might be a cause-oriented and effective therapy for patients with SCC.

## Materials and Methods

### Cell culture and proliferation assay

The procedure has been described previously [14, 37]. Briefly, the HeLa cell line was purchased from DS Pharma Biomedical (Suita, Osaka, Japan) and was cultured in α-MEM (Gibco-BRL, Gaithersburg, MD) with 10% fetal bovine serum (FBS; SAFC Bio Sciences, Brooklyn, Victoria, Australia). We simultaneously received the cell authenticity report (KBN0198) on July 7, 2014 from the Japan Collection of Research Bioresources (JCRB) showing that the cell line was not misidentified or contaminated. Cell proliferation in response to EMP9, EMP1, and rhEpo was examined using a detection kit (MK420, Takara, Otsu, Shiga, Japan). The EMP9 and EMP1 were synthesized by the Peptide Institute (Minoo, Osaka, Japan); the rhEpo was a gift from Kyowa Hakko Kirin Co., Ltd. (Tokyo, Japan).

### Mice and in vivo tumor formation

The procedure has been described previously [14]. A total of 15 male nude mice (BALB/cA Jcl-nu/nu, CLEA, Tokyo, Japan) that were 6–8 weeks old were subcutaneously injected with HeLa cells. When the tumors developed palpable, we measured the tumor size twice per week. Two mice with approximately 6 × 7 × 7 mm tumors were sacrificed at 13 days after palpable tumor recognition, and the tumors were extirpated without any treatment (HeLaX-NT). Other two mice did not show any tumors at 13 days after the cell injection; they were excluded from the study. For the other mice, when the tumors were approximately 6 × 7 × 5 mm at 20 days after palpable tumor recognition, we intraperitoneally injected EMP9 or saline (Otsuka Pharmaceutical, Tokyo, Japan) 3 times per day at 1-hr intervals every three days with or without phentolamine-HCl (ph) (LKT Laboratories, Inc., St. Paul, MN) 1 hr before injection of EMP9 for 2 days (Table 1). As HeLa cells express α1A adreno-receptor and the expression levels are upregulated in the xenografts [36], we injected its antagonist, ph. We used the following doses of EMP9: 9 (E9-9), 4.5 (E9-4.5), and 2.25 (E9-2.25ph) mg (Table 1). Accordingly, we designated the experimental tumor models as HeLaX-E9-9, HeLaX-E9-4.5, HeLaX-E9-2.25ph, and two types of controls HeLaX-S (saline-treated) and HeLaX-NT (non-treated). Two weeks after the initiation of the treatments, the mice were sacrificed using intraperitoneal injection of 0.1 ml Somnopentyl (Kyoritsu Seiyaku, Tokyo, Japan) followed by 0.8 ml of blood draw from the heart, and then the tumors were extirpated. The animal experiments described in this paper were approved by the Animal Care and Use Committee of Kinki University and performed according to the guidelines set by Animal Care Handling at Kinki University Faculty of Medicine.

**Table 1:** Suppression of viability of HeLa cell line treated with EMP9 alone or EMP9 plus ph in nude mice.

Experimental groups (no. of mice used)	Treatment with EMP9 or EMP9 and ph^(a)^			Extirpation after last injection (day)	Mean tumor growth at extirpation (mg)^(c)(d)^	Tumor viability dead/alive^(f)^ areas (%)
	Injection^(b)^	Total dose (mg)	Injection^(h)^, frequency 3/day × days			
HeLaX-E9-9 (3)	EMP9	9	0, 1, 2 × 3	10	182.9	41.2 / 58.8*
HeLaX-E9-4.5 (3)	EMP9	4.5	0, 1, 2 × 3	10	-96.7	39.7 / 60.3*
HeLaX-E9-2.25ph (3)	EMP9+ph	EMP9 2.25	0, 1, 2 × 1; 1, 2, 3 × 2	10	225.2	44.6 / 55.4**
		Ph 0.5	24, 48			
HeLaX-S (2)	saline		0, 1, 2 × 3	10	361.3	35.2 / 64.8
HeLaX-NT (2)	none			^(e)^	148.7	25.4 / 74.6

Abbreviations: (a) phentolamine, (b) Beginning at 20 days after palpable tumor recognition, (c) Average growth indicates the value at extirpation subtracted those just before treatment, (d) In non-treated mice, values at extirpation subtracted those at palpable tumor recognition, (e) The tumors developed enough to treat, then the tumors were extirpated at 13 days after palpable tumor recognition, (f) Dead and alive areas were determined as described in "Materials and Methods", (h) hour.

*, and **, indicate significant differences between non-treated and each group by Chi Square's test, at P<0.05, and P<0.01.

### Macroscopic determination of dead and alive areas

The procedure has been described previously [38]; this macroscopic viability assay is based on the mitochondrial dehydrogenase activity to detect viable cells. Briefly, central portions of the extirpated tumors (HeLaXs) were selectively extracted and cut transversely into slices 1 mm thick. Then, median two or three slices were chosen for this assay. The slices were incubated with 2% formazan in PBS for 10 min at 37°C in darkness and then fixed in 4% neutral-buffered formalin to stabilize the red color. These samples were used for the determination of dead (white) and alive (red) areas using Image J (http://rsb.info.nih.gov/ij/). The remaining tissue samples were used for the following immunohistochemical analyses or biochemical analyses.

### Immunohistochemistry

The procedure has been described previously [6, 13, 39]. Briefly, the tissue samples described above were fixed with Zamboni solution, treated in 25% sucrose for cryoprotection, frozen, and then cut into slices 7 μm thick. We used the following antibodies according to the previous studies: anti-EpoR antibody (1:100) [39]; anti-ε (1:125) and anti-γ (1:125) [5]. The following antibodies were purchased from Santa Cruz Biotechnology (Dallas, TX): anti-FcRII/III (CD16/32; 1:500), anti-IFNγ (1:2000), anti-HIF-1α (1:50), anti-COL5A_1_ (1:100), anti-nNOS (1:250), anti-iNOS (1:250), anti-eNOS (1:250), anti-CD38 (1:200), and anti-α (1:100). The following primary antibodies were purchased from Biolegend (San Diego, CA): anti-MHC II (1:10), anti-mouse-NK-1.1 (1:200), anti-mouse CD11b (1:100), anti-mouse CD11c (1:200), and anti-mouse CD19 (1:200). The other antibodies were purchased as follows: anti-Mb (1:100: Abcam, Cambridge, UK), anti-Ki67 (1:100, Dako, Glostrup, Denmark), anti-cytochrome C (1:100, Sigma-Aldrich, St. Louis, MO), anti-F4/80 (1:250, AbD Serotic, Oxford, U.K.), anti-iNOS (1:1000, R&D Systems, Minneapolis, MN). The double staining was performed using anti-α (1:500), anti-ε (1:500), anti-γ (1:500), or anti-Mb (1:250, Abcam) with Alexa Fluor 546 (Invitrogen) or 488 (Molecular Probe, Leiden, Netherland) as the secondary antibodies [40] and Dapi (Vetashield, Vector Laboratories, Burlingame, CA) for the nuclear staining.

### RT-PCR and quantitative RT-PCR analyses

We performed RT-PCR to confirm the expression of the non-erythroid heme synthase, δ-aminolevulinate synthase (ALAS-N), and the absence of the erythroid specific δ-aminolevulinate synthase (ALAS-E). The primer sequence of ALAS-N and ALAS-E, the procedures for the DNA synthesis, and the PCR conditions were described previously [5].

For the quantitative RT-PCR analysis, we extracted total RNA from the HeLaXs using Trizol (Invitrogen, Carlsbad, CA). A High-Capacity cDNA Reverse Transcription Kit (Applied Biosystems, Foster City, CA) was used to synthesize cDNA. Then, the expression of the transcripts of Epo, EpoR, nNOS, iNOS, eNOS, Hbα (α), Hbε (ε), Hbγ (γ), Hbβ (β), Mb and ALAS-N was measured by real-time quantitative RT-PCR with an ABI PRISM 7900HT sequence detection system (Applied Biosystems). All the PCR reactions as well as the primers and probes, except for NOSs and ALAS-N, were reported previously [5]; the primers and probes for nNOS (Hs 00167223_m1), iNOS (Hs 01075529_m1), eNOS (Hs 01574659_m1) and ALAS-N (Hs 00963534_m1) were supplied by TaqMan Gene Expression Assays (Applied Biosystems). The data were obtained in triplicate for each sample. The expression levels of each mRNA are shown as relative contents to the respective expression levels of 18S rRNA mRNA.

### Western blotting

The procedure has been described previously for extracting the tissue lysates, separation of the membrane fractions from the tissue lysates, and subsequent immunoblotting [5]. We purchased the following antibodies from Cell Signaling Technology (Danvers, MA) for intracellular signaling detection: anti-AKT, anti-pAKT, anti-MAPKs, anti-pMAPKs, anti-STAT5, and anti-pSTAT5. Each lane was loaded with 40 μg of protein, and the bands were detected using a luminoimage analyzer system (LAS-4010, GE Healthcare Life Sciences, Piscataway, NJ). The band intensity was quantified with Image J software.

### Immune cell population in the xenografts

The sections stained with the FITC-labeled anti-mouse CD11b, anti-mouse NK 1.1, anti-mouse CD38 and anti-mouse CD11c antibodies for the macrophages, NK cells, B-cells and DC, respectively, were counted using 50 definite areas (0.036 mm^2^) in the intra-tumor regions of the HeLaXs under a fluorescent microscope (ECLIPSE equipped Y-FL, Nikon, Tokyo, Japan).

### Capillary density

The sections were stained with anti-collagen V (COL5A_1_), which is synthesized by endothelial cells and traceable underneath the endothelium. Under a 200 x magnified frame of 0.036 mm^2^, cross- and longitudinally sectioned capillaries were counted in 50 definite areas including the degenerating and non-degenerating lobules from five sections each of the HeLaXs.

### Proliferating and perforated cells and cells in apoptosis

We counted the tumor cells with an immunoreactive Ki67 nucleus, those with pores in the sections stained with hematoxylin and eosin, and those with positive cytochrome C grains [41, 42] in definite areas (0.036 mm^2^) of 7–10 sections, 8–15 sections, and 20 sections, respectively.

### Statistical analysis

The procedure has been described previously [43]. Briefly, statistical significance of differences between two groups was determined by Student's t-test; one-way analysis of variance with Holm’s post-hoc test was used for multiple group comparison. Chi-square test was used to compare the incidence. All data were analyzed by R Environment (R Project). P<0.05 was considered to be statistically significant.

## Results

### EMP9 suppresses growth of HeLa cells *in vitro*

The mean tumor growth 14 days after the treatment was as follows: 182.9 mg for 9 mg EMP9, -96.7 mg for 4.5 mg EMP9, 225.2 mg for 2.25 mg EMP9 plus 0.5 mg ph, and 361.3 mg for vehicle control (Table 1). A reduction in tumor growth was seen only in the mice treated with EMP9 at 4.5 mg although it was not significant. However, the mean tumor growth in the mice treated with EMP9 was smaller than that in the saline-treated mice. The hematocrit values were 49.2 ± 2.8%, 42.6 ± 9.1%, 50.0 ± 1.4% and 49.3% in tumor-bearing mice exposed to 9 and 4.5 mg of EMP9, 2.5 mg of EMP9 and 0.5 mg of ph and saline, respectively. These results indicate no harmful effect of EMP9 on the hematopoiesis in the hosts.

The total viable regions of three experimental xenografts (the HeLaX-E9s) were significantly smaller than those of the HeLaX-NTs (Table 1, P<0.05, P<0.01) without dose dependency for EMP9. No significant differences were observed in the total viable areas between the saline-exposed xenografts (HeLaX-Ss) and the HeLaX-NTs (Table 1). These data suggest that EMP9, but not saline, suppressed the viability of the HeLaXs in nude mice in this study.

**Fig 1:**
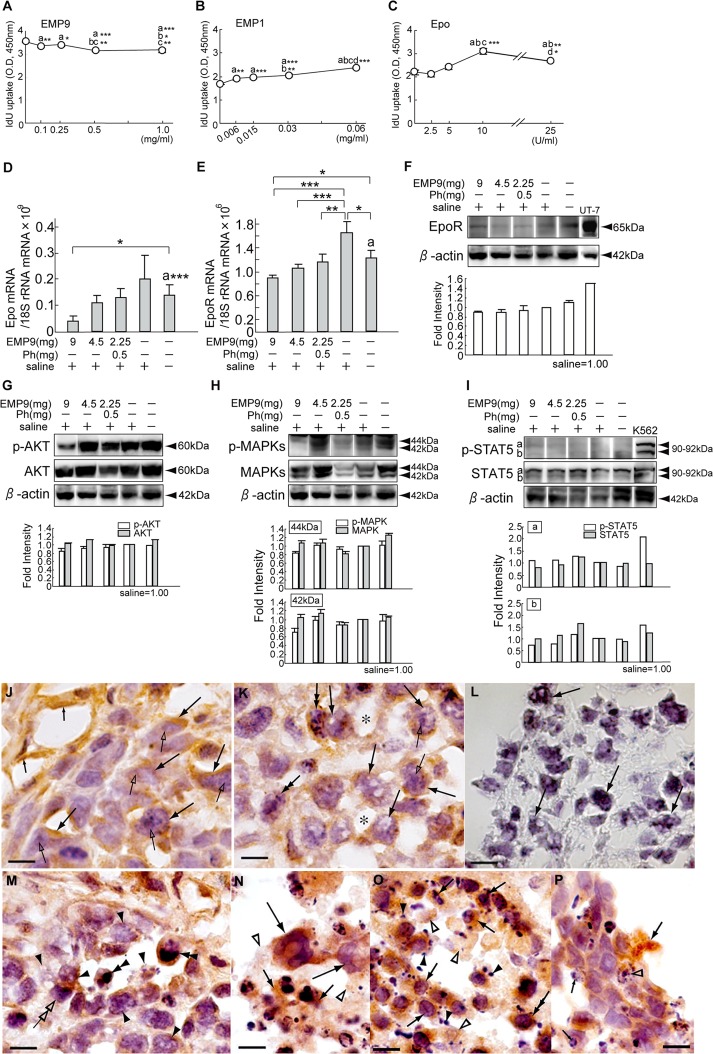
EMP9 suppresses HeLa cell growth in vitro and inhibits Epo-EpoR signaling in the xenografts. A—C. HeLa cells were exposed to EMP9 (A) or EMP1 (B) four times at 1-h intervals for 24 hr (A, B), or to rhEpo (C) four times at 24-h intervals for 4 days as described previously [14]. The letter on the dot means significant differences from the value at 0 mg or 0 U/ml (a), 2nd dosage (b), 3rd dosage (c) and 4th dosage (d). *, ** and *** indicate significant difference from the value for each letter by ANOVA-test, at *, P<0.05, **, P<0.01, ***, P<0.001, respectively. D—E. The levels of Epo mRNA (D) and EpoR mRNA (E) in the experimental and control xenografts. *, ** and *** indicate significant differences from average levels of Epo mRNA and EpoR mRNA by Student's t-test and by ANOVA-test, respectively, at *, P<0.05, **, P<0.01, ***, P<0.001, respectively. The letter a means significant differences between the average levels of Epo and EpoR mRNA in the HeLaX-NTs by Student's t-test, ***, P<0.001 (a). F—I. Western blottings of EpoR protein (F), pAKT and AKT (G), pMAPKs and MAPKs (H), and pSTAT5 and STAT5 (I) in both experimental and control xenografts. Fold intensity of each band shows adjusted value by the density of HeLaX-Ss as 1.0. J—L. Immunoreactivity to EpoR antibody in HeLaX-Ss (J) and HeLaX-E9-9s (K). Arrows point to tumor cells (J, K, L), small arrows, capillary endothelial cells (J), open arrows, nucleolus (J, K), double arrows, chromatin condensed nuclei (K) and asterisks, capillary lumens (K). L. Negative control for EpoR antibody stained with rabbit IgG in section of HeLaX-E9-9s. J—L, Scale bar, 10 μm. M—P. Expression of EpoR in immune cells at the degenerating regions. M: EpoR is recognizable in macrophages engulfing dying particle (double arrowheads) or contacted with (double open arrow) dying tumour cell with pores (arrowheads). N: Huge macrophages (long arrows) and presumptive NK cells (arrows) show EpoR in their cytoplasm. Remnant dead cells (open arrowheads). O: Presumable NK cell (arrows), and B cell (double arrow) show EpoR in their cytoplasm. Remnant of dead cells (open arrowheads). Note many calcium deposits (arrowheads) beneath these immune cells. P: EpoR expression in dendrites of presumable dendritic cells (arrow). Note many chromatin condensation in dying tumor cells (open arrowhead) and immune cells (small arrows). M—P, Scale bar, 10 μm.

The HeLa cells responded to all the doses of EMP9 (Fig 1Aa, P<0.05, P<0.01, P<0.001) and significantly to 0.5 mg/ml of EMP9 (Fig 1Ab, P<0.01; Fig 1Ac, P<0.01). They decreased in a weak dose-dependent manner up to 0.5 mg/ml of EMP9 (Fig 1Ab, P<0.01; Fig 1Ac, P<0.01), and the fold-decrease ranged from 0.88 to 0.96 of that of the control. The HeLa cells responded to all the doses of EMP1 (Fig 1Ba, P<0.01; Fig 1Bd, P<0.001), and they increased in response to rhEpo (Fig 1Ca, 1Cb and 1Cc, P<0.001; Fig 1Ca and 1Cb, P<0.01). The fold increase ranged from 1.10 to 1.43 in EMP1 and 0.96 to 1.41 in rhEpo compared with the respective control. These data suggest that EMP9 could suppress the growth of HeLa cells *in vitro*.

### EMP9 suppresses Epo-EpoR signaling in the tumor sites *in vivo*

The Epo mRNA levels showed a dose-dependent decrease in response to EMP9 without significant differences. However, the levels were significantly lower in the HeLaX-E9-9s than in the HeLaX-NTs (Fig 1D, P<0.05). The EpoR mRNA levels were significantly lower in the HeLaX-E9s than in the HeLaX-Ss (Fig 1E, P<0.01, P<0.001). The EpoR mRNA levels of HeLaX-NTs were lower than those of the HeLaX-Ss (Fig 1E, P<0.05) and higher than those of the HeLaX-E9-9s (Fig 1E, P<0.05). The overall EpoR mRNA level was approximately 10^3^ times higher than that of the Epo mRNA in the HeLaXs (Figs 1D and 1E).

The immunoblots revealed expression of the EpoR protein in all the samples, showing weaker intensities in the HeLaX-E9s than in the controls (HeLaX-Ss and HeLaX-NTs) (Fig 1F). These bands were not identified with those for HSP70 (Cell Signaling Technology, data not shown). All the samples constitutively expressed pAKT (Fig 1G), pMAPKs (Fig 1H), and pSTAT5 (Fig 1I). The band intensity for pAKT was weaker in the HeLaX-E9s than in the controls (Fig 1G). However, that for pMAPKs and pSTAT5b was weaker than in the controls in the HeLaX-E9-9s and HeLaX-E9-2.25phs (Fig 1H) and in the HeLaX-E9-9s and HeLaX-E9-4.5s (Fig 1I), respectively.

The following immunohistological results were observed. In the HeLaX-Ss, the tumor cells and capillary endothelial cells showed strong EpoR immunoreactivity (Fig 1J). In the HeLaX-E9s, the tumor cells and capillary endothelial cells did not show results similar to those of the controls, and instead, the cells showed perforation, small or large pores, in the tumor cell body (Fig 1K), concomitant with the loss of endothelial cells (Fig 1K). Negative immunoreactivity is shown in the section stained with rabbit IgG (Fig 1L). Further, phagocytic and cytotoxic activities were detectable in macrophages (Fig 1M and 1N), many immune cells (Fig 1O) and DCs (Fig 1P). These immune cells showed immunopositivity of EpoR in their cytoplasm (Fig 1M–1P). Thus, Epo-signal appears to be involved in tumorigenesis of HeLa cells and in activation of immune cells, and that EMP9 could reduce the expression of Epo and EpoR mRNA and suppress Epo-EpoR signaling in HeLaXs, leading to a loss of viability of the tumor cells and capillary endothelial cells.

### Degenerating patterns in HeLaXs

**Fig 2:**
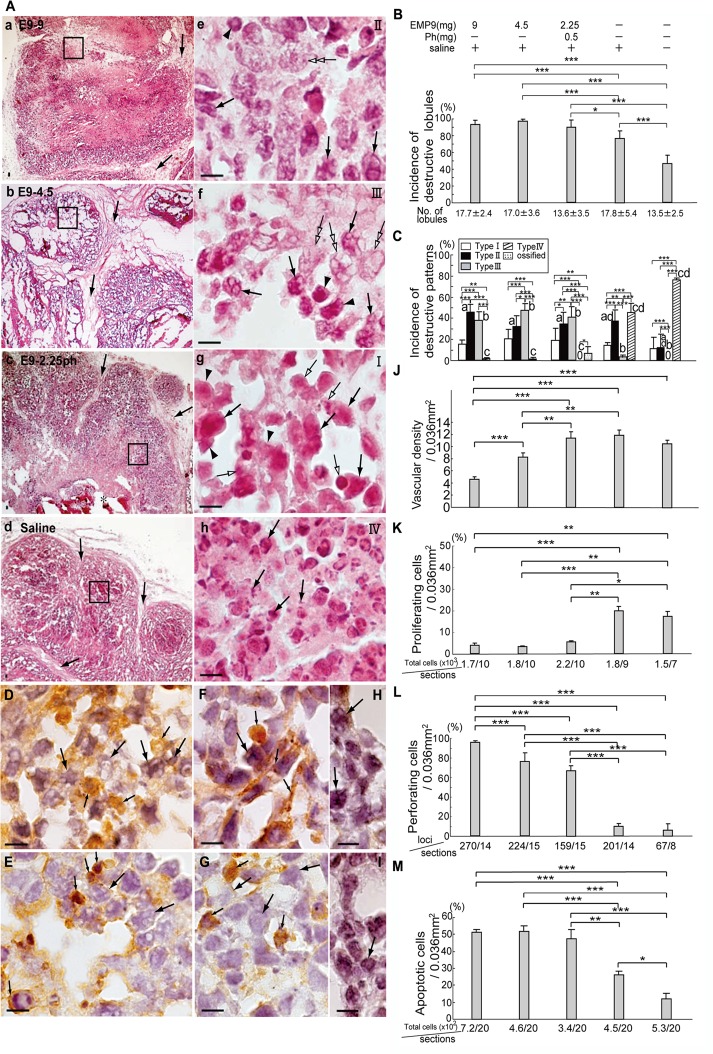
How xenografts were destroyed and resolved through EMP9 treatment in nude mice. A. Various types of degenerating lobule (a—d), and their magnified figures (e—h). Arrows in a—d indicate intervening fibrous cords; Three types of characteristic degenerating region are shown in a and e as Type II, in b and f as Type III, in c and g as Type I and in d and h as Type IV (necrotic figures). Arrows in e—g indicate tumor cells, arrowheads and open arrows point to presumptive macrophage and NK cells, respectively. Double open arrows in e and f point to dying tumor cells with large pores. Arrows in h point to pyknotic nuclei or nuclear fragments. Asterisk in c shows calcified masses. a—h, Scale bar, 10 μm. Incidence of destructive lobules (B) and each degenerating type (C) in HeLaX-E9s, HeLaX-Ss and HeLaX-NTs. *, ** and ***, and a—d indicate significant differences by Chi-square test, at *, P<0.05, **, P<0.01, ***, P<0.001, respectively. a, b, c and d indicate significant difference from the value of NT (a), from the value of HeLaX-Ss or HeLaX-NTs (b), from the value of HeLaX-Ss or HeLaX-NTs (c), and between the same letter (d), at P<0.001. D. Type II region of HeLaX-E9-9s showing macrophages (small arrows) attached to tumor cells with pores (arrows). E. Type III region of HeLaX-E9-9s showing NK cells (small arrows) attached to tumor cells (arrows). F, G. Non-degenerating region of HeLaX-Ss showing macrophages (small arrows, F) and NK cells (small arrows, G) near tumor cells (arrows, F, G). H, I. Negative control for anti-F4/80 antibody stained with rat-IgG in HeLaX-E9-9s (H) and for anti-mouse NK1.1 antibody stained with rat IgG in HeLaX-E9-4.5s. Arrows point to tumor cells (H) and to presumptive NK cell (I). Scale bar, 10 μm. J. Vascular density in each HeLaXs. Individual vessels were counted as described in "Materials and Methods". **and *** indicate significant differences by ANOVA-test, at **, P<0.01 and ***, P<0.001, respectively. K—M. Population of proliferating tumor cells (K), perforating tumor cells (L) and apoptotic cells (M). *, **and *** indicate significant differences by Chi-square test, at *, P<0.05, **, P<0.01 and ***, P<0.001.

The histopathological examinations revealed that the HeLaXs showed lobule-like structures (Fig 2Aa, 2Ab, 2Ac and 2Ad). Nearly all the lobules showed weak or strong destructive features, including degeneration and/or necrosis, and the incidence of destructive lobules was significantly higher in the HeLaX-E9s than in the HeLaX-Ss (Fig 2B, P<0.05, P<0.001). We categorized the mild to severe degeneration into three types, Type I, Type II, and Type III, with necrosis as Type IV (Fig 2Ag, 2Ae, 2Af and 2Ah), and summarized the incidence of the four types in the xenografts in Fig 2C. Type III occurred significantly higher in the HeLaX-E9s than in the controls (P<0.001); HeLaX-E9-4.5s showed the highest incidence of Type III among the HeLaX-E9s (P<0.001). The occurrence of Type IV, signifying necrosis, was extremely higher in the controls than in the HeLaX-E9s (P<0.001).

In the Type II and Type III regions, macrophages stained with anti-F4/80 (Fig 2D) and NK cells stained with anti-NK 1.1 antibodies (Fig 2E) attached to the perforating tumor cells and appeared to be eliminating the dying tumor cells in the HeLaX-E9-9s (Fig 2D and 2E), whereas in the HeLaX-Ss, macrophage (Fig 2F) and NK cells (Fig 2G) were in contact with the tumor cells with or without pores (Fig 2F and 2G). Negative immunoreactivity to anti-F4/80 and anti-NK 1.1 is shown in the sections stained with rat IgG and mouse IgG, respectively (Fig 2H and 2I). These findings suggest that EMP9 induces the degeneration of HeLaXs, particularly in HeLaX-E9-4.5s, concomitant with the recruitment of activated immune cells to kill the tumor cells and eliminate the dying components, leading to destruction of the xenografts.

### EMP9 inhibits angiogenesis in the tumor sites

The vascular densities were significantly lower in the HeLaX-E9-9s and HeLaX-E9-4.5s than in the HeLaX-Ss (Fig 2J, P<0.001, P<0.01), and a dose-dependent decrease was detectable in the HeLaX-E9s (Fig 2J). There were significantly fewer tumor cells with positive Ki67 nucleus staining with the anti-Ki67 antibody in the HeLaX-E9s than in HeLaX-Ss (Fig 2K, P<0.01, P<0.001) and HeLaX-NTs (Fig 2K, P<0.05, P<0.01). There were significantly more tumor cells with pores in the HeLaX-E9s than in the controls (P Fig 2L, P<0.001). The tumor cells that expressed cytoplasmic cytochrome C stained with the anti-cytochrome C antibody increased significantly in the HeLaX-E9s compared with the controls (Fig 2M, P<0.001, P<0.01). These data suggest that EMP9 destroys the vascular nets, suppresses tumor cell growth and induces perforation and apoptosis of tumor cells, leading to destruction of xenografts.

### EMP9 induces HIF-1α expressions in the tumor sites

**Fig 3:**
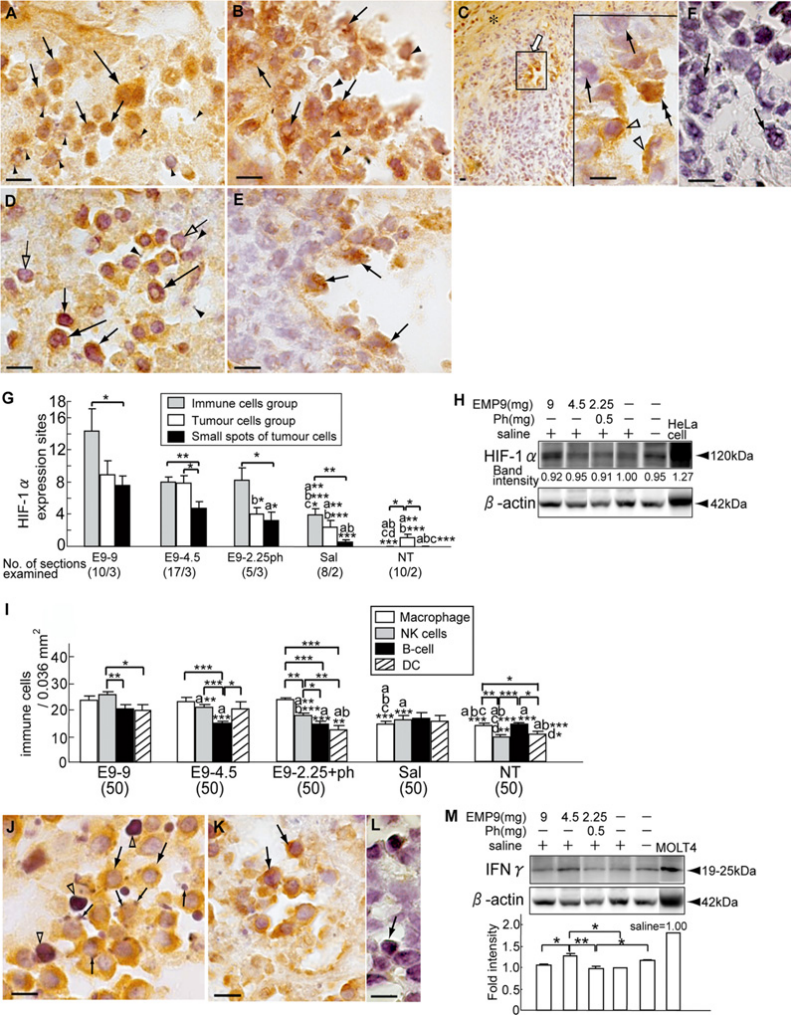
Histopathological features in xenografts. Prominent HIF-1α expression in degenerating (A, B) and non-degenerating region (C) of HeLaX-E9-9s, and weak expression in HeLaX-Ss (D, E). Long arrows in A, D point to macrophages, arrows in B, C, E, to tumor cells, open arrows in D, presumptive NK cells with negative immunoreactive HIF-1α, arrowheads in A, D, calcium deposits, arrowheads in B, immune cells, open arrowheads in inlet of C, presumptive macrophages, double arrows in inlet of C, tumor cell with positive HIF-1α. F. Negative control for anti-HIF-1α in the HeLaX-E9-9s stained with rabbit IgG shows tumor cells with pores (arrows). A—F, Scale bar, 10 µm. G. Incidence of cell groups expressing HIF-1α at three different sites. Number in parentheses indicate the number of sections out of number of xenografts examined. Significant differences were examined by Student’s t-test. The letters a, b, c and d indicate significant difference between the value of HeLaX-Ss and the value at E9-9 (a), E9-4.5 (b), E9-2.25ph (c) and Sal (d), respectively. *, P<0.05, **, P<0.01, ***, P<0.001. H. Immunoblotting of HIF-1α protein in xenografts and HeLa cells. I. Macrophages, NK cells, B-cells and DCs were counted as described in "Materials and Methods". Average number of respective immune cells in HeLaX-E9s, HeLaX-Ss and HeLaX-NTs. *, **and *** indicate significant differences among four immune cells and between respective cells in HeLaX-E9s and HeLaX-Ss, or in HeLaX-E9s and HeLaX-NTs by Student's t-test, at *, P<0.05, **, P<0.01, ***, P<0.001, respectively. The letter on each bar means significant differences from the value of HeLaX-E9-9s (a), HeLaX-E9-4.5s (b), HeLaX-E9-2.25phs (c) and HeLaX-Ss (d) by Student's t-test. *, P<0.05, **, P<0.01, ***, P<0.001. The number in parentheses means the counted number of definite area (0.036 mm2) in the intra-tumor regions of the HeLaXs. J—K. Expression of IFNγ in NK cells in the HeLaX-E9-2.25phs (J) and HeLaX-Ss (K). Arrows point to NK cells with IFNγ immunopositivity (J, K), small arrows, calcium droplets (J) and open arrowheads, dead calcified cells (J). L. Negative control for IFNγ antibody. Arrow points to presumptive NK cells. J—L, Scale bar, 10 μm. M. Immunoblot of IFNγ in HeLaXs. *and ** indicate significant differences in the level of the intensity among experimental and control xenografts by ANOVA-test, at *, P<0.05, **, P<0.01, respectively.

Because HeLa cells show the band for HIF-1α under hypoxia and an intensively strong band under anoxia in western blot analysis [35], we confirmed the expression in the HeLaXs. The degenerating regions of the HeLaX-E9s showed crowding of the majority of the immune cells (Fig 3A), and the tumor cells (Fig 3B) had nuclei with immunoreactive HIF-1α; additionally, there were spots in a few cells in the non-degenerating regions (Fig 3C). Cells with HIF-1α immunoreactivity were less numerous in the controls than in the HeLaX-E9s (Fig 3D and 3E). The negative control for HIF-1α is shown in Fig 3F. The number of sites, including the cells expressing HIF-1α in each HeLaX, is shown (Fig 3G). The HIF-1α expression sites in the immune cells and tumor cells were significantly more numerous in the HeLaX-E9s than in each control (P<0.05, P<0.01, P<0.001). The immunoblots confirmed the HIF-1α protein in the HeLaXs and HeLa cells (Fig 3H).

### Population of the immune cells and the relevant cytokines expression

Macrophages were significantly more numerous in the HeLaX-E9s than in the controls (Fig 3I, P<0.001) and were comparable among the HeLaX-E9s (Fig 3I). The number of NK cells was highest in the HeLaX-E9-9s among the HeLaX-E9s (Fig 3I, P<0.01, P<0.001) and significantly higher than in the controls (Fig 3I, P<0.001); in the HeLaX-E9-2.25phs, the number was significantly smaller than in the macrophages (P<0.01). No significant differences in the number of B cells and DCs were seen between the HeLaX-E9s and HeLaX-Ss (Fig 3I).

IFNγ was discernible in the cytoplasm of the activated NK cells in the degenerating regions of the HeLaX-E9-9s, with calcified dying cells and small or large calcium (Ca) deposits (Fig 3J). Additionally, in the HeLaX-Ss, NK cells with IFNγ were detectable; however, no calcified cells or Ca deposits were seen, suggesting that they had weak activation (Fig 3K). The negative control for the IFNγ antibody is shown in the section stained with rat IgG (Fig 3L). Western blotting clarified that the intensity of IFNγ was significantly higher in HeLaX-E9-4.5s (P<0.05) than in HeLaX-Ss (Fig 3M). In addition to IFNγ, IL-1α and TNFα were detectable in the immune cells and stromal cells in HeLaX-E9s; however, no significant difference in the expression was detectable from the HeLaX-Ss in their immunoblotting data. These findings suggest that EMP9 exposure augments the infiltration of macrophages and NK cells to activate them to secret cytokines in the xenografts, leading to the upregulation of their cytotoxic effects.

### Expression of NOSs

**Fig 4:**
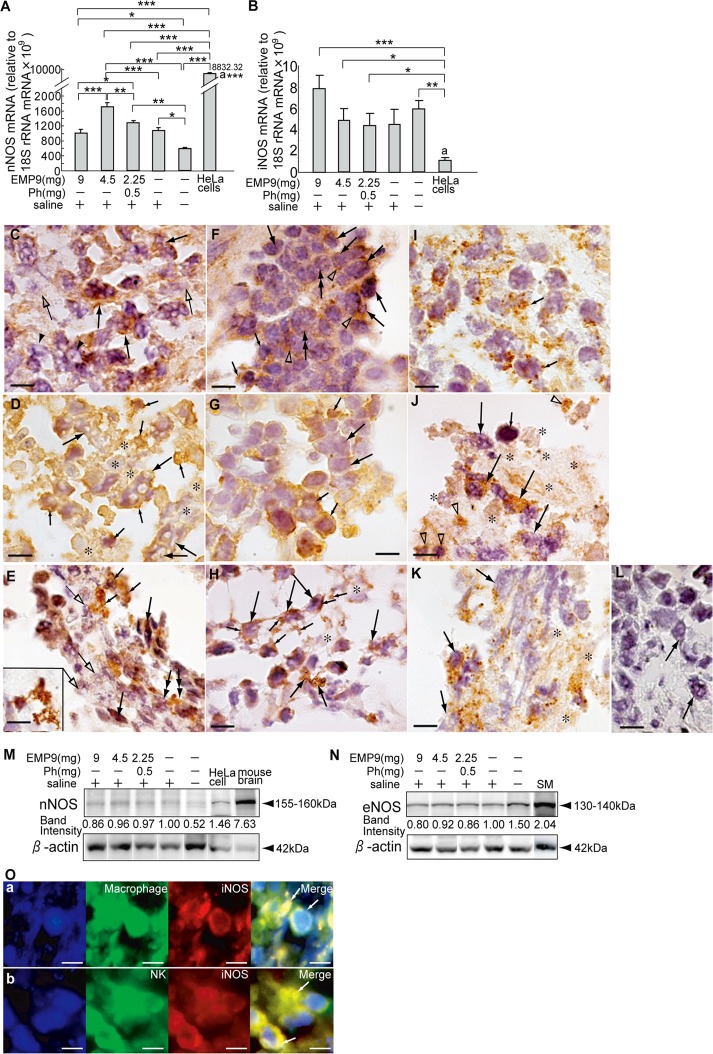
Expression of NOSs in xenografts of HeLa cells. A, B. Expression levels of nNOS mRNA (A) and iNOS mRNA (B) in HeLaXs. *, **and *** indicate significant differences between experimental and control xenografts, and between HeLaXs and HeLa cells by ANOVA-test (A) and by Student's t-test (B), at *, P<0.05, **, P<0.01, ***, P<0.001, respectively. The letter a means significant differences between average levels of nNOS mRNA and iNOS mRNA in HeLa cells by Student's t-test, at P<0.001. C—H. Respective NOS expression, nNOS (C, F), iNOS (D, G) and eNOS (E, H), in HeLaX-E9s (C, D, E) and in HeLaX-Ss (F, G, H). Arrows in C, D, F, G and H point to tumor cells with pores (C, D) or without pores, and in E, fibrous cells; middle arrows in H, indicate DCs; small arrows in D, F and G point to presumptive immune cells, and in E and H, processes of DCs. Open arrows in C and E point to dying cells; open arrowheads in F point to immunopositive nNOS; and asterisks in D and H indicate remnant of dying cells. I—K. Corresponding regions to C, D and E stained with anti-cytochrome C indicating apoptotic death. Small arrows in I and long arrows in J and K point to tumor cells in apoptotic death. Open arrowheads in J point immune cells in apoptotic death. Asterisks in J, K indicate remnant of dying cells. L. Negative control for NOSs antibodies stained with rabbit IgG in HeLaX-E9-9s. Arrow points to tumor cells. M, N. Immunoblots for nNOS (M) and eNOS (N) proteins in HeLaX-E9s and their controls. Each band intensity is shown adjusted value by the density of β-actin in the HeLaX-Ss as 1.0. SM indicates safety margin of lung cancer which is the remainder used on 2010 [40]. O. Localization of iNOS in macrophages (arrows) in HeLaX-E9-9s (a) and in NK cells (arrows) in HeLaX-E9-4.5s (b).

In cervical cancer, the expression of inducible NO synthase (iNOS) has been reported to be a useful prognostic marker [22, 23]. We examined the expression of three types of NOSs. In parental HeLa cells, transcripts for nNOS and iNOS mRNA were detected; however, transcripts for eNOS were not detected (Fig 4A and 4B). The expression level of nNOS mRNA was highest in the HeLa cells of all of the HeLaXs (Fig 4A, P<0.001). HeLaX-E9-4.5s showed the highest expression compared with the other HeLaX-E9s (Fig 4A, P<0.01, P<0.001) and the controls (Fig 4A, P<0.001). The expression level of iNOS mRNA was significantly higher in the HeLaX-E9s than in the HeLa cells (Fig 4B, P<0.05, P<0.001); the highest level was observed in the HeLaX-E9-9s (Fig 4B, P<0.001). However, there were no significant differences in the expression levels among the HeLaXs.

Immunopositivity to nNOS, iNOS, and eNOS varied among the HeLaX-E9s (Fig 4C–4E) and HeLaX-Ss (Fig 4F–4H). In HeLaX-E9-9s, nNOS immunoreactivity was prominently detectable in the perforated tumor cells (Fig 4C), whereas in HeLaX-Ss, immunoreactivity was detectable in the intercellular spaces, suggesting that the expression is on the cell surface (Fig 4F). iNOS was strongly detected on the cell membrane of the tumor cells with many pores and in the immune cells attached to tumor cells with large pores in the HeLaX-E9-4.5 (Fig 4D). In the HeLaX-Ss, immune cells with immunoreactive iNOS were attached to the tumor cells without pores (Fig 4G). Pores observed in the tumor cells with immunoreactive nNOS and iNOS showed a different outline, with a circle with a distinct edge or an irregular pore, respectively (Fig 4C and 4D), suggesting the different sites of NO ejection from the cell surface or in the cytoplasm, respectively. eNOS was observed in the immune cells, especially in the DCs, in both the HeLaX-E9s and HeLaX-Ss (Fig 4E and 4H). The dying features of the respective regions of HeLaX-E9s (Fig 4I–4K) indicated apoptotic death of the perforated tumor cells expressing nNOS (Fig 4I), a cytotoxic appearance with remnants and/or apoptotic death in both iNOS- (Fig 4J) and eNOS-expressing regions (Fig 4K). Staining specificity to the three antibodies is demonstrated in the section stained with rabbit IgG (Fig 4L).

Western blots demonstrated the expression of nNOS and eNOS in all of the samples, including HeLa cells. The band intensities among the HeLaX-E9s were weaker in the HeLaX-E9-9s than that in the HeLaX-Ss (Fig 4M and 4N). iNOS was confirmed in the macrophages and NK cells in the HeLaX-E9-9s and HeLaX-E9-4.5s (Fig 4O).

These histopathological data suggest that NO generated from nNOS in the tumor cells and iNOS in the macrophages, NK cells and HeLa cells might act as an auto-cytotoxic and cytotoxic and cytostatic factor in tumor cells.

### Expression of hemoglobins in the tumor sites

**Fig 5:**
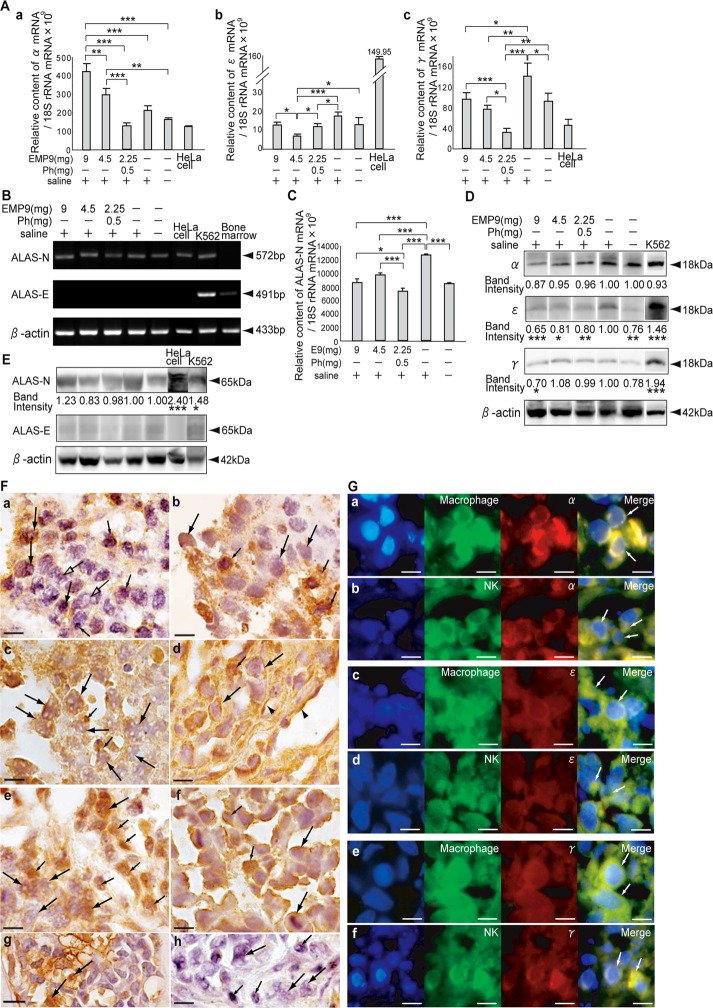
Expression of hemoglobins in HeLaXs. A. The expression levels of mRNAs for α (a), ε (b) and γ (c) in the experimental and control xenografts and in the HeLa cells. *, ** and *** indicate significant differences by ANOVA-test, at *, P<0.05, **, P<0.01 and ***, P<0.001, respectively. B. Expression of ALAS-N mRNA in HeLaXs and HeLa cells by RT-PCR analysis. Control for K562, erythroleukemia cell line, expresses both ALAS-N and ALAS-E mRNA; bone marrow expresses only ALAS-E mRNA. C. The expression levels of ALAS-N mRNA in the HeLaX-E9s, HeLaX-Ss and HeLaX-NTs. * and *** indicate significant difference by ANOVA-test, at *, P<0.05 and ***, P<0.001, respectively. D, E. Western blot results of α, ε and γ (D), and ALAS-N and ALAS-E (E) in the HeLaXs. Band intensity shows adjusted value by the density of β-actin of the HeLaX-Ss as 1.0. *, **and *** indicate significant difference from HeLaX-Ss in ε, γ and ALAS-N by ANOVA-test, at *, P<0.05, **, P<0.01 and ***, P<0.001, respectively. F. Expression of hemoglobins in HeLaX-E9s (a, c, e) and HeLaX-Ss (b, d, f). In F, arrows and small arrows point to immunopositive α (a, b), ε (c, d) or γ (e, f) in tumor cells and immune cells, respectively. Arrowheads point to capillary endothelial cells with immunopositive ε (d). Positive control of α is shown in host bone marrow, arrows point to developing red blood cells (g). Negative immunoreactivity to ε and γ is shown in section stained with rabbit IgG in HeLaX-E9-4.5s. Arrows point to tumor cells, small arrows, immune cells (h). G. Localization of α in macrophages (arrows) of HeLaX-E9-9s (a) and in NK cells (arrows) in HeLaX-E9-4.5s (b). Localization of ε in macrophages (arrows) of HeLaX-Ss (c) and in NK (arrows) cells of HeLaX-E9-9s (d). Localization of γ in macrophages (arrows) of HeLaX-Ss (e) and in NK cells (arrows) of HeLaX-E9-9s (f). E—F, all bar, 10 μm.

In all of the xenografts, α, ε and γ globin mRNAs were seen, whereas β globin mRNA was not seen (Fig 5A); these mRNA levels are shown in Fig 5A. The expression levels of α globin mRNA in the HeLaX-E9-9s was the highest among the HeLaX-E9s and HeLaX-Ss (Fig 5Aa, 5Ab and 5Ac), suggesting that α globin pairs with ε or γ globin, forming α_2_ε_2_ and α_2_γ_2_, embryonic and fetal Hbs. The expression levels of ε globin mRNA in the HeLaX-E9-4.5s and HeLaX-E9-2.25phs were significantly lower than in the HeLaX-Ss (Fig 5Ab, P<0.001, P<0.05), and those of γ globin mRNA were significantly lower in the HeLaX-E9s than in the HeLaX-Ss (Fig 5Ac, P<0.05, P<0.01, P<0.001). Because the heme molecules for these Hbs and Mb were synthesized by the rate-limiting isozyme for the nonerythroid heme, non-erythroid δ-aminolevulinate synthase (ALAS-N), we confirmed ALAS-N mRNA and its expression levels in the HeLaXs and HeLa cells (Fig 5B and 5C). The levels showed significantly lower in the HeLaX-E9s than in the HeLaX-Ss (Fig 5C, P<0.001).

Western blots of these globins showed lower densities in the HeLaX-E9-9s than in the HeLaX-Ss (Fig 5D). The expression of ALAS-N proteins was higher in the HeLaX-E9-9s and lower in the HeLaX-E9-4.5s and HeLaX-E9-2.25phs than in the HeLaX-Ss (Fig 5E).

Immunopositive α (Fig 5Fa and 5Fb), ε (Fig 5Fc and 5Fd) and γ globins (Fig 5Fe and 5Ff) were detectable in the tumor cells with pores (Fig 5Fa, 5Fc and 5Fe) and in the activated immune cells (Fig 5Fa, 5Fc and 5Fe) of the experimental xenografts; they were also detectable in the tumor cells without pores (Fig 5Fb, 5Fd and 5Ff) and in the immune cells of the control xenografts (Fig 5Fb, 5Fd and 5Ff). Furthermore, immunoreactive ε globin was detectable in the capillary endothelial cells (Fig 5Fd). The positive control for the α globin antibody is shown in the host bone marrow stained with the α antibody (Fig 5Fg), and the negative controls for the ε and γ globin antibodies are shown in the sections stained with rabbit IgG (Fig 5Fh).

Co-localization of α, ε and γ globin in the macrophages and in the NK cells was confirmed in the HeLaX-E9s and HeLaX-Ss (Fig 5G). These co-expression data indicate that tumor cells and immune cells express Hbs in the embryonic, α_2_ε_2_ and fetal, α_2_γ_2_ forms.

### Expression of Mb in the HeLaXs

**Fig 6:**
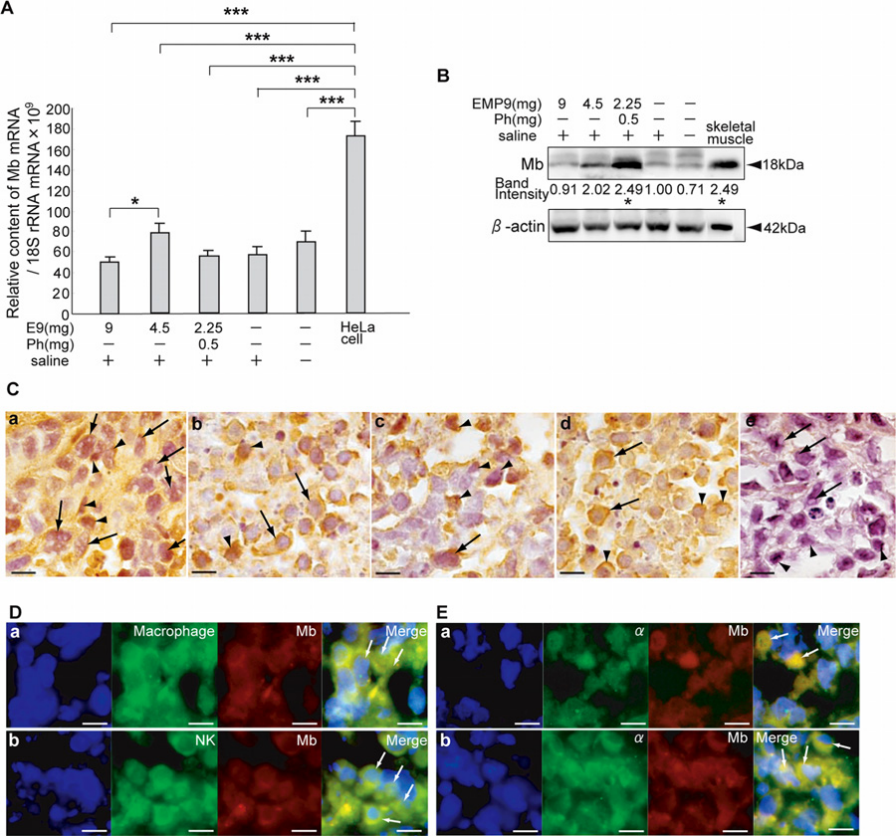
Expression of Mb in HeLaXs. A. The expression levels of Mb mRNA in the experimental and control xenografts and in the HeLa cells. * and *** indicate significant difference by ANOVA test, at *, P<0.05 and ***, P<0.001, respectively. B. Western blotting results of the Mb in the HeLaXs. Band intensity shows adjusted value by the density of β-actin of the HeLaX-Ss as 1.0. *, indicates significant difference from HeLaX-Ss by ANOVA test, at *, P<0.05. C. Expression of immunopositive Mb in HeLaX-E9-s9 (a, b) and HeLaX-Ss (c, d). Arrows in a, c and e point to tumor cells, and in b and d, probable NK cells. Arrowheads in a, c and e, point to immune cells, and b and d, presumptive B cells.

No significant differences in the expression levels of Mb mRNA were detected between the HeLaX-E9s and HeLaX-Ss; however, the levels between the HeLaX-E9-9s and HeLaX-E9-4.5s showed a significant difference (Fig 6A, P<0.05). A western blot of Mb showed a lower density in HeLaX-E9-9s and a higher density in the HeLaX-E9-4.5s and HeLaX-E9-2.25phs compared with the HeLaX-Ss (Fig 6B).

Negative immunoreactivity to Mb is shown in section stained with mouse IgG in HeLaX-E9-4.5s (e). D. Localization of Mb in macrophage in HeLaX-E9-9s (a) and NK cells in HeLaX-E9-2.25phs (b). E. Co-localization of α and Mb in presumptive tumor cells (arrows) in HeLaX-E9-4.5s (a) and in presumptive NK cells (arrows) in HeLaX-Ss (b). C—E, all bar, 10 μm.

Immunopositive Mb was detectable in the tumor cells with pores and in the activated immune cells in the HeLaX-E9s (Fig 6Ca and 6Cb); in the HeLaX-Ss, there were fewer tumor cells and immune cells with positive Mb immunoreactivity compared with the HeLaX-E9s (Fig 6Cc and 6Cd). The negative control for the Mb antibody was observed in the section stained with mouse IgG (Fig 6Ce). Localization of Mb in the macrophages and NK cells (Fig 6D) and co-localization of α globin and Mb in the presumptive tumor cells (Fig 6Ea) and presumptive immune cells (Fig 6Eb) were confirmed. These data indicate that tumor cells and immune cells express embryonic and fetal Hbs and monomeric Mb.

## Discussion

This study demonstrates a novel role of Epo in cancer cells. Epo is involved in embryonic (α_2_ε_2_) and fetal (α_2_γ_2_) Hb and Mb syntheses in the xenografts of HeLa cells, which suggests that these hemoproteins are reserved for the constant demands for O_2_ in the HeLa cells. The blockade of Epo signaling using an Epo antagonist EMP9 suppressed the expression of EpoR, ε and γ globin with heme synthetic enzyme ALAS-N, which was accompanied by intracellular Ca deposits and extracellular Ca^2+^ release in the HeLaX-E9s. Moreover, consinstent with the previous studies [6, 13, 36], the treatment induced apoptosis of the tumor cells and inhibited angiogenesis in the xenografts. These findings were associated with the enhanced expressions of nNOS in the HeLaX-E9s and iNOS in innate immune cells such as macrophages and NK cells in the tumor sites. Given that the treated HeLaX-E9s exhibited a substantial number of perforations on the cell surface, the tumors were damaged by both the nNOS-induced NO production in the HeLaX-E9s as well as the iNOS-induced NO production in the innate immune cells.

We observed that the EMP9 treatment inhibited the AKT-pAKT, MAPKs-pMAPKs, and STAT5-pSTAT5 signaling pathways (Fig 1G–1I). In this regard, we have examined several cancer cell lines (DLD1, HepG2, P39, T98G, PC-3, G361, SCH), of which expression levels of Epo and EpoR are already known, to investigate whether rhEpo triggered and EMP9 inhibited tyrosine phosphorylation of STAT5 [37]. As a result, tumor growth rate in response to rhEpo stimulation corresponded to the level of the constitutive tyrosine phosphorylation of STAT5. Moreover, EMP9-suppressed growth depends inversely on the levels of Epo secretion, with less Epo indicating greater suppression of growth. The 24-hr secretion amounts of Epo in 22 cell lines ranged from 0.04 mU to 13.4 mU/mg of protein, and the level of Epo in the HeLa cells was approximately 0.18 mU. These *in vitro* data might strengthen the finding in the current *in vivo* study.

Physiologically, Epo-EpoR binding induces an increase in intracellular free Ca [Ca^2+^i] through a voltage-independent Ca^2+^ channel, which is known to prerequisite for erythroid proliferation and differentiation [44, 45]. In contrast, in our tumor model, Ca deposits were frequently detected in the HeLaX-E9s (Fig 1N and 1O, Fig 2Ac and 2C, Fig 3A). Here, high Ca^2+^ is known to bind preferentially to phosphate ions to form calcium deposits. These findings indicate altered intracellular Ca distribution in the Epo/EpoR-expressing tumor cells and capillary endothelial cells. That is, a voltage-dependent Ca^2+^ channel promoted an extreme increase in [Ca^2+^i] in these cells. As a result, excessive cytoplasmic Ca^2+^ influx led to cell death and/or release into extracellular space. In addition, higher extracelluar Ca^2+^ concentration induced the formation and release of calreticulin-containing azur granules form NK cells and to induce nNOS and eNOS activation in these cells (Fig 3J). In parallel, the increased extracelluar Ca^2+^ is known to promote lyposomal activities in neutorophils [46].

Based on our data (Fig 5Ab and 5Ac, Fig 6A), ectopic Mb appeared to compensate the ability of Hb α_2_ε_2_ and α_2_γ_2_ to reserve/storage O_2_. Ectopic Mb is known to be expressed in some cancers [47] and co-expressed with α_1_ globin in MCF-7 and HeLa cells [48]. Mb plays a certain role in oxygen storage and diffusion. Moreover, it protects the cells from deep hypoxia by scavenging NO and/or reactive oxygen species (ROS) [49–51]. In the HeLaXs, Mb, α, ε and γ globin were co-expressed (Fig 5G, Fig 6E). Moreover, the Mb expression levels were upregulated in HeLaX-E9-4.5s compared with that in the HeLaX-E9-9s (Fig 6A, P<0.05) along with a higher density of the Mb protein than that of the HeLaX-Ss (Fig 6B). In the HeLaX-E9-4.5s, the ε globin mRNA level was the lowest among the HeLaX-E9s (Fig 5Ab, P<0.05) and HeLaX-Ss (Fig 5Ab, P<0.001) concomitantly the lowest protein levels (Fig 5D, P<0.05, P<0.01, P<0.001). The decreased O_2_ concentration induced by the α_2_ε_2_ concentration in HeLa cells might trigger the upregulation of Mb expression. Among the levels of α, ε and γ globin mRNA, those of γ globin mRNA were significantly downregulated in all HeLaX-E9s but those of ε globin mRNA showed significantly low in the HeLaX-E9-4.5s and HeLaX-E9-2.25phs (Fig 5Ab and 5Ac). Although the ε protein levels decreased in a dose-dependent manner in the HeLaX-E9s, α and γ showed the lowest protein levels in the HeLaX-E9-9s (Fig 5D), suggesting the downregulation of α_2_ε_2_ and α_2_γ_2_ production through EMP9 exposure. Thus, it is likely that in the HeLaXs, EMP9 inhibited both synthesis of embryonic and fetal Hbs particularly that of embryonic Hb, ε, in the HeLaXs.

The oxygen binding curve of Mb is rectangular hyperbola, and that of HbE, HbF or HbA blood is sigmoidal [52]. The dissociation or binding of O_2_ and/or NO in cells is different among these hemoproteins. The binding capacity to O_2_ is highest in Mb among these four, and HbE has the higher capacity than HbF and HbA. Irreversible alteration of the composition of these hemoproteins in response to EMP9 treatment (Fig 5A, 5C, 5D and 5E) implies the suppression of O_2_ binding capacity of the tumor cells, which led to decreased O_2_ reserves and in turn induced detrimental damage and finally death. This finding is supported by the previous observation that rhEpo injection improved tumor oxygenation in R3230 rodent mammary carcinoma [53]. There, these ectopic hemoprotein appear to contribute to the promotiong and survival of cancer cells by supplying O_2_ individually.

Epo signaling appears to function similarly between erythroid progenitor cells and the Epo/EpoR-expressing HeLa cells in the xenografts; it stimulates tumor cell proliferation and production of embryonic and fetal Hbs with monomeric Mb hemoprotein to survive. A blockade of Epo signaling destroyed the xenografts by inhibition of tumor growth, angiogenesis and survival through disruption of the harmonized hemoprotein syntheses in the tumor cells, leading to the loss of protection against O_2_, NO or ROS toxicity. In addition, macrophages and NK cells with intense HIF-1α expression recruited significantly more in the degenerating foci of the HeLaX-E9s. The treated tumor cells exhibited a substantial number of perforations on the cell surface, which indicates that the tumors were damaged by both the nNOS-induced NO production in the tumor cells as well as the iNOS-induced NO production in the innate immune cells. Although the host's immune cells expressed the immunoreactive EpoR (Fig 1M–1P), they might escape from the effects of EMP9 because of their short life span and enourmous number of their replacement, 0.7 day and 2.4 × 10^7^ per day, respectively in mouse white blood cells [54]. This study clarified that a cause-oriented therapy might be useful for various cancers that express Epo signaling.
